# Plug Closure of an Ascending Aortic Graft Anastomotic Pseudoaneurysm After Mediastinitis

**DOI:** 10.1016/j.atssr.2026.01.012

**Published:** 2026-01-29

**Authors:** Keiichi Ishida, Yoshiaki Katada, Hitoshi Nakanowatari, Ikuko Shibasaki, Yoshihito Irie

**Affiliations:** 1 Department of Cardiovascular Surgery, Iwaki City Medical Center, Iwaki, Japan; 2 Department of Radiology, Tokyo Medical University Ibaraki Medical Center, Ibaraki, Japan

## Abstract

Ascending aortic pseudoaneurysms are rare but high-risk complications after cardiac surgery. Whereas open repair remains standard, transcatheter closure offers a less invasive option for high-risk patients. We report the case of a 50-year-old man in whom an ascending aortic graft anastomotic pseudoaneurysm developed 10 months after aortic valve and ascending aortic replacement complicated by mediastinitis. As redo surgery carried a significant risk, an Amplatzer vascular plug II was deployed across the anastomotic defect with a disk-to-disk configuration, achieving complete exclusion. Follow-up computed tomography at 3 and 6 months revealed progressive shrinkage of the pseudoaneurysm, supporting plug closure as a feasible alternative.

Ascending aortic pseudoaneurysms (AAPs) are rare but life-threatening complications that occur in 0.5% of cardiac surgical cases, most often after aortic root surgery or coronary artery bypass grafting.^1^ They typically develop at aortic cannulation sites, saphenous vein graft sites, or aortotomy sites and may be manifested either early or several years after surgery.^1^ AAPs carry significant mortality risk, with early mortality approaching 20% and yearly mortality rates up to 40%.^1^ Although open surgical repair remains the standard treatment, redo sternotomy can be hazardous in high-risk patients. Accordingly, transcatheter closure has emerged as a less invasive alternative for selected cases.^2^^,^^3^

Herein, we report a graft anastomotic pseudoaneurysm successfully treated by percutaneous closure with an Amplatzer vascular plug II (AVP II) in a patient with postoperative mediastinitis, highlighting technical considerations and the feasibility of plug closure in complex cases.

A 50-year-old man presented with fever persisting for several days, 10 months after mechanical aortic valve and ascending aortic graft replacement complicated by *Staphylococcus capitis* mediastinitis, which was successfully treated with surgical drainage, omental flap transposition, and intravenous administration of vancomycin. Laboratory tests revealed a serum C-reactive protein level of 18.1 mg/L. Contrast-enhanced computed tomography (CT) revealed a mediastinal hematoma and an AAP (Figure 1A). Retrospective review of a CT scan obtained 4 months earlier revealed the same pseudoaneurysm with leakage of contrast material from the proximal anastomotic site (Figure 1B). He was urgently admitted with an AAP diagnosis; intravenous administration of vancomycin was started, and blood cultures were persistently negative. Transesophageal echocardiography revealed no vegetations, prosthetic dysfunction, or aortic root abscess. Inflammatory markers gradually declined, and repeated CT at 4 days revealed no change in pseudoaneurysm size.

**Figure 1:**
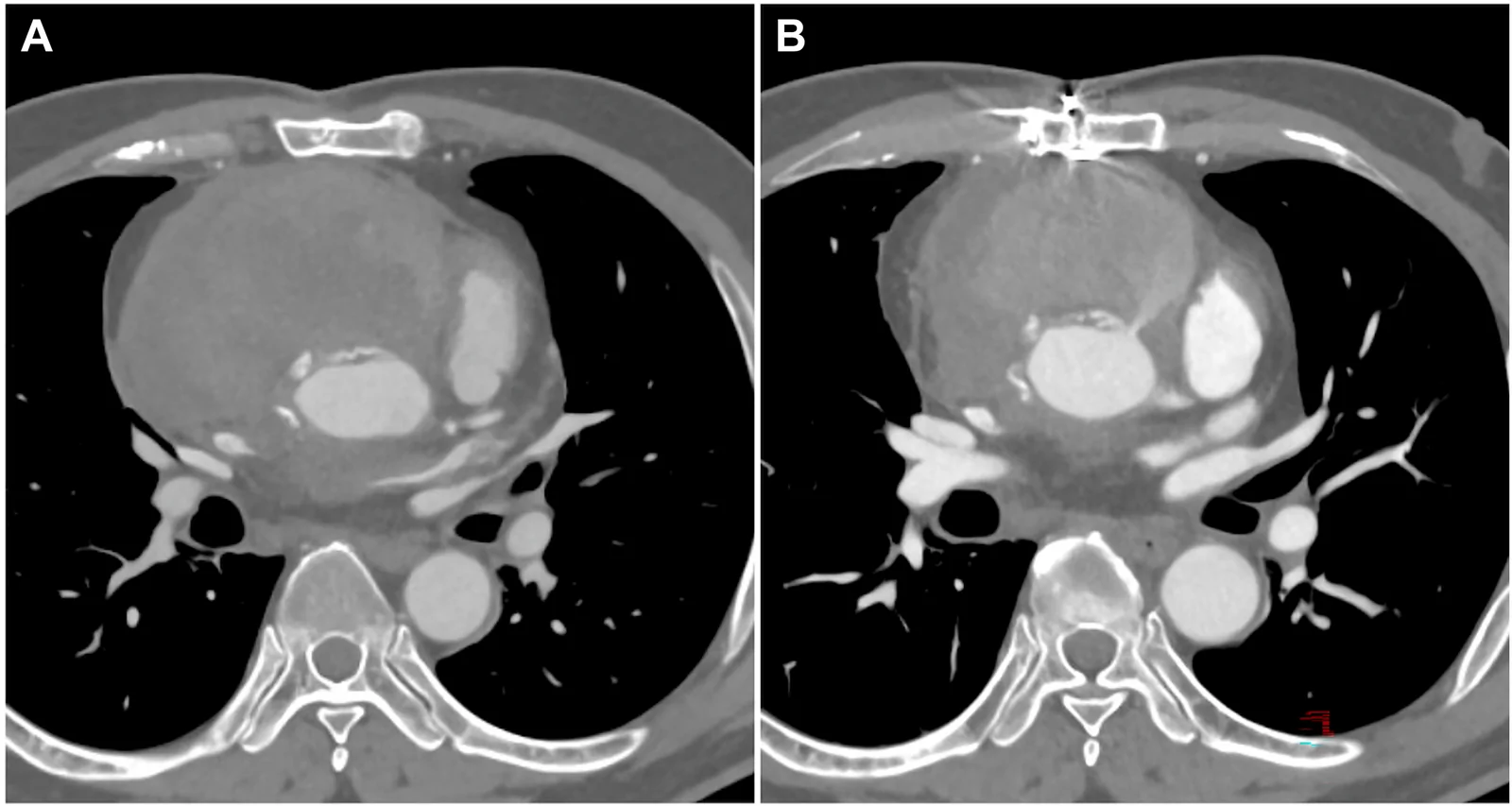
(A) Admission contrast-enhanced computed tomography demonstrates a mediastinal hematoma and large ascending aortic pseudoaneurysm (107 × 59 mm). (B) Retrospective review of a computed tomography scan obtained 4 months earlier depicts a preexisting pseudoaneurysm with evident extravasation of contrast material originating from the proximal anastomotic site.

Japan SCORE2 predicted an operative mortality of 10.6% and a combined major morbidity and mortality risk of 37.6%. Although redo graft offered definitive repair, the risk was deemed prohibitive owing to prior mediastinitis, omental flap transposition, and ongoing warfarin therapy. The absence of active infection and the chronic nature of the pseudoaneurysm supported reconsideration of open surgery. Endovascular plug closure was deemed feasible, and the patient strongly preferred a minimally invasive approach.

Under general anesthesia, a 6F 90-cm guiding sheath (Destination guiding sheath; Terumo Corporation) was advanced from the right brachial artery into the aortic arch. A 5F diagnostic catheter (Cobra type; Medkit Co, Ltd) was used to engage the pseudoaneurysm entry near the proximal anastomosis of the graft. The pseudoaneurysm was delineated by contrast-enhanced angiography (Figure 2A). A 0.035-inch Radifocus guidewire (Terumo Corporation) was advanced into the pseudoaneurysm. The 5F catheter was also advanced, but the guiding sheath could not pass the narrow entry. Therefore, an Amplatz Super Stiff guidewire with a 1-cm soft tip (Boston Scientific) was gently curved, allowing successful advancement of the guiding sheath into the cavity through the 5F catheter. A 12-mm AVP II (Abbott Cardiovascular) was deployed with 2 disks positioned within the pseudoaneurysm and the final disk positioned within the ascending aorta to straddle and occlude the entry site (Figure 2B). The device was released after confirming stability and complete occlusion on angiography. Final angiography revealed complete exclusion of the pseudoaneurysm without residual flow (Figure 2C).

**Figure 2:**
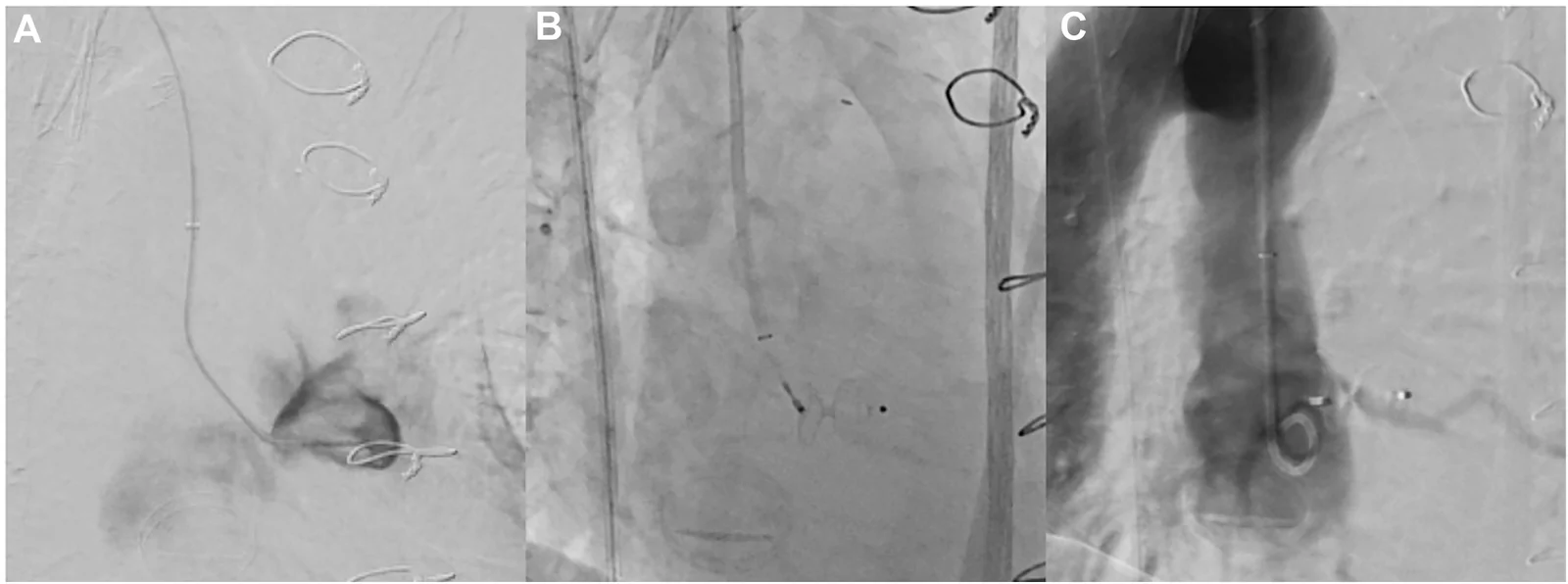
(A) Pre-embolization angiography reveals opacification of the pseudoaneurysm cavity, with a jetlike reflux of contrast material into the ascending aorta on forceful injection. (B) Deployment of the third disk of the Amplatzer vascular plug II within the ascending aortic lumen achieved stabilization across the entry defect. (C) Post-embolization angiography confirms complete flow cessation into the pseudoaneurysm sac.

Postprocedural CT confirmed correct device position with no leak (Figure 3A). Recovery was uneventful, and the patient was discharged on postoperative day 11. At follow-up, he remained clinically stable with no signs of infection, and CT scans at 3 and 6 months demonstrated progressive shrinkage of the pseudoaneurysm (Figures 3B, 3C).

**Figure 3:**
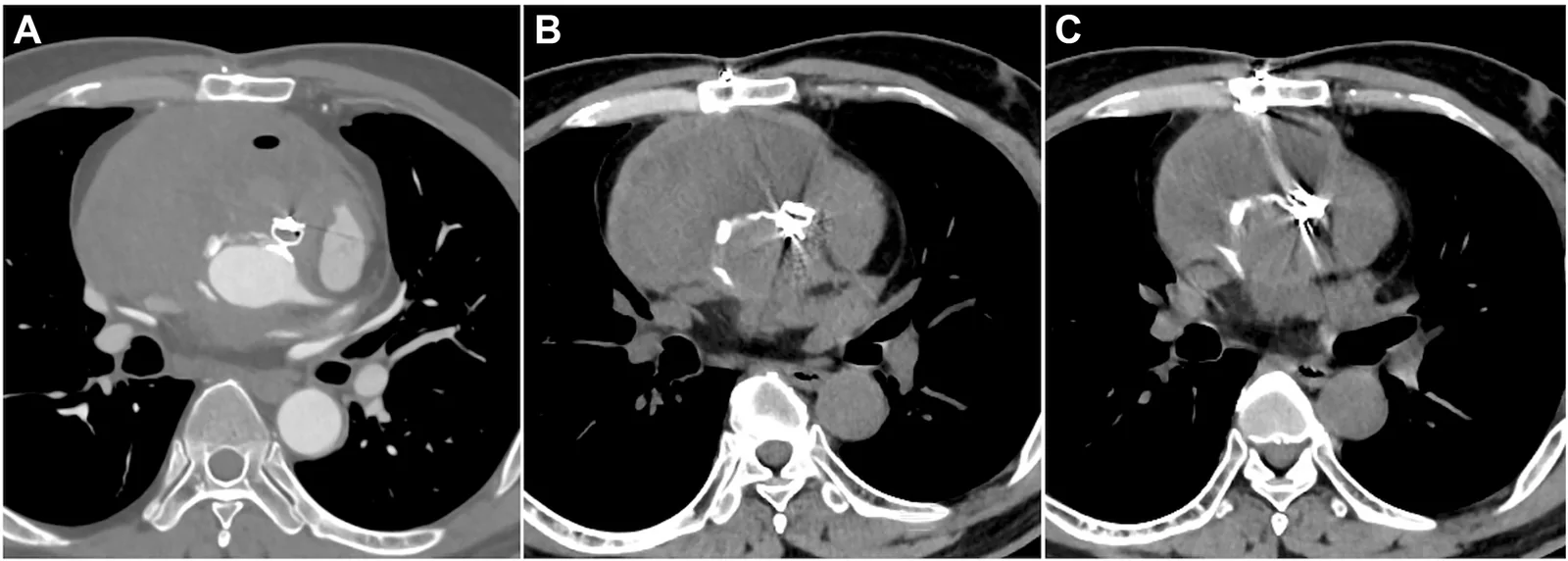
Serial computed tomography images after plug closure. (A) Immediately after the procedure, no residual leak or device migration is observed; the pseudoaneurysm measured 108 × 56 mm. (B) At 3 months, an interval reduction of the pseudoaneurysm (101 × 48 mm) is noted. (C) At 6 months, further shrinkage to 85 × 40 mm is evident, demonstrating progressive involution over time.

## Comment

Various plug closure techniques for postoperative AAPs have been described. For example, multiple case reports have described successful percutaneous closure with Amplatzer devices, including septal occluders and vascular plugs.^4^^,^^5^ Patel and coworkers^5^ provided a comprehensive literature review of the percutaneous closure technique. Moreover, Tang and coworkers^2^ demonstrated the value of multimodality imaging for managing complex cases. However, complications remain possible. Noble and coworkers^6^ reported device embolization 27 days after the procedure with an Amplatzer muscular ventricular septal defect occluder. Katada and colleagues^3^ described fatal hemoptysis with plug closure of wide-neck pseudoaneurysms after transcatheter aortic valve implantation, highlighting the limitation of plug closure in certain anatomies.

In this case, device selection was based on several anatomic and technical considerations. As the shunt hole was approximately 2 mm, minimizing mechanical stress during catheter and sheath manipulation was essential. Although a 6F guiding sheath was ultimately required, compatibility with a 5F system was preferred to reduce entry-site load. Surgical felt adjacent to the shunt hole suggested that a device covering this reinforced area would provide more secure occlusion; thus, a 12-mm AVP II was selected. The second and third disks of the plug were positioned to “sandwich” the aortic wall, ensuring stable disk-to-disk apposition. To avoid high-velocity aortic flow affecting the disk, which is an important issue particularly relevant in the ascending aorta, the third disk was intentionally placed within the aortic lumen to alleviate hemodynamic stress on the defect.

Serial imaging demonstrated progressive shrinkage of the pseudoaneurysm during 6 months. Reduction was more pronounced between 3 and 6 months than immediately after the procedure. The early phase likely reflects thrombosis within the pseudoaneurysm cavity and AVP II–induced mechanical flow cessation, whereas neointimal formation across the device may contribute to more complete and durable closure over time. Katada and colleagues^7^ reported that the surface of the device demonstrates favorable neointimal coverage when positioned within the aorta, suggesting that biologic healing processes progressively reinforce mechanical occlusion and may ultimately result in complete sealing of the shunt hole.

In conclusion, for high-risk patients or those for whom redo sternotomy is prohibitive, plug closure represents an effective and minimally invasive alternative for managing ascending AAPs. This case adds to growing evidence that endovascular plug closure is a viable therapeutic option in carefully selected patients.
